# Implications of the school-household network structure on SARS-CoV-2 transmission under school reopening strategies in England

**DOI:** 10.1038/s41467-021-22213-0

**Published:** 2021-03-29

**Authors:** James D. Munday, Katharine Sherratt, Sophie Meakin, Akira Endo, Carl A. B. Pearson, Joel Hellewell, Sam Abbott, Nikos I. Bosse, CMMID COVID-19 Working Group, Rosalind M. Eggo, David Simons, Kathleen O’Reilly, Timothy W. Russell, Rachel Lowe, Quentin J. Leclerc, Jon C. Emery, Petra Klepac, Emily S. Nightingale, Matthew Quaife, Kevin van Zandvoort, Gwenan M. Knight, Thibaut Jombart, C. Julian Villabona-Arenas, Eleanor M. Rees, Charlie Diamond, Megan Auzenbergs, Graham Medley, Anna M. Foss, Georgia R. Gore-Langton, Arminder K. Deol, Mark Jit, Hamish P. Gibbs, Simon R. Procter, Alicia Rosello, Christopher I. Jarvis, Yang Liu, Rein M. G. J. Houben, Stéphane Hué, Samuel Clifford, Billy J. Quilty, Amy Gimma, Damien C. Tully, Fiona Yueqian Sun, Kiesha Prem, Katherine E. Atkins, Jacco Wallinga, W. John Edmunds, Albert Jan van Hoek, Sebastian Funk

**Affiliations:** 1 Centre for Mathematical Modelling of Infectious Diseases, London School of Hygiene and Tropical Medicine, London, UK; 2 Department of Infectious Disease Epidemiology, London School of Hygiene and Tropical Medicine, London, UK; 3 Centre for Global Health, Usher Institute, College of Medicine and Veterinary Medicine, University of Edinburgh, Edinburgh, UK; 4 National Institute for Public Health and the Environment (RIVM), Bilthoven, The Netherlands; 5 Department of Biomedical Data Sciences, Leiden University Medical Centre, Leiden, The Netherlands

**Keywords:** Computational models, SARS-CoV-2, Viral infection, Epidemiology

## Abstract

In early 2020 many countries closed schools to mitigate the spread of SARS-CoV-2. Since then, governments have sought to relax the closures, engendering a need to understand associated risks. Using address records, we construct a network of schools in England connected through pupils who share households. We evaluate the risk of transmission between schools under different reopening scenarios. We show that whilst reopening select year-groups causes low risk of large-scale transmission, reopening secondary schools could result in outbreaks affecting up to 2.5 million households if unmitigated, highlighting the importance of careful monitoring and within-school infection control to avoid further school closures or other restrictions.

## Abstract (web summary)

Many countries have closed schools as part of their COVID-19 response. Here, the authors model SARS-CoV-2 transmission on a network of schools and households in England, and find that risk of transmission between schools is lower if primary schools are open than if secondary schools are open.

## Introduction

School closures are one of many non-pharmaceutical interventions that can be employed during epidemics of droplet infections, such as influenza, to reduce transmission, and can be highly effective^1–3^. However, there are substantial societal and economic costs associated with closing large numbers of schools, such as limiting children’s access to education and requiring caregivers to stay at home (impacting on household income and on economic activity)^4–8^, which can affect more economically deprived households most^9^. As with any public health intervention, it is important for policy makers to balance the public health benefits of school closures with the associated economic and social impact. To do this effectively, clear understanding of the relative benefit of closing schools and therefore the potential impact of reopening is required.

School closures were introduced as a central component of the response to the COVID-19 outbreak in many countries around the world^10^. The UK closed all schools on 23 March 2020 to all but the children of essential workers and the most vulnerable. Schools in England remained closed to the majority of students until the beginning of the academic year (September 2020). Although reported cases of COVID-19 continue to be low amongst school-aged children, the role of children in transmitting COVID-19 remains unclear^11–15^, and studies in the UK show comparable prevalence in children and adults^16^. The contribution of transmission within schools to transmission within the community is still uncertain and may have been an important factor in the resurgence of disease in the population in recent months.

Notwithstanding the poorly quantified risk, over the summer there was growing concern regarding the potential impact of prolonged closures on the wellbeing of the population at large^17^. A report from the Royal Society voices concerns that maintaining widespread closures does not just pose a risk to children’s wellbeing in the immediate term but may also have long term consequences for the skill level of the future workforce and therefore economic growth of the UK^18^. These concerns ultimately led to the decision to reopen schools to all years in September 2020.

The potential contribution of schools to transmission is twofold: firstly, the number of potentially infectious contacts increases through children mixing in schools. Secondly, transmission within schools can facilitate transmission between households, and households with multiple school-aged children attending different schools may act as a route for transmission between schools. This second impact can be considered as a network of schools and households linked by pupils. While strict stay-at-home orders (so-called lockdowns) as implemented in many countries had the aim of removing the links in the network such that chains of infection could not progress beyond individual households, reopening schools has the potential of reconnecting households with each other such that longer chains of infection can arise.

Here, we investigate the connectivity of the school and household network and, consequently, on the potential for schools to contribute to transmission by allowing chains of transmission to infect many households. We quantify this by presenting the potential reach of an outbreak among families with school-aged children, under the assumption that children are effective at transmitting the virus. We do so by using a large data set of household addresses of school children in England to quantify the probability of transmission via pupils who reside in a common household as the edges on a network of schools. We use this framework to analyse the potential for these links between schools to form large networks of infectious contact and therefore large outbreak clusters within the school-age population and their household members.

## Results

### Networks of household-based contact between schools

**Fig. 1:**
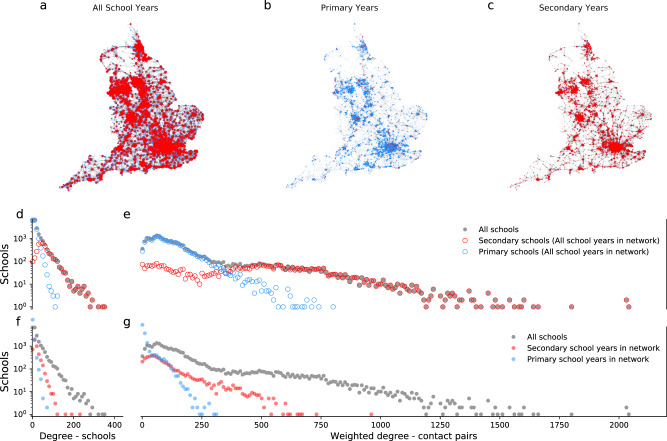
School contact networks. Networks of contact through households between 21,608 state-funded schools in England plotted by location. **a** Network with all school years in attendance. **b** Network with only primary school years in attendance. **c** Network with only secondary school years in attendance. Nodes show schools with size determined by the weighted degree of the node (number of unique contact pairs with any other school). Edge widths that indicate the number of unique contact pairs between the schools the edge connects. Red nodes show secondary schools (mean age ≥11 years), blue nodes show primary schools (mean age <11 years). Followed by degree distributions of the networks of contact through households. **d** A histogram of the number of schools connected by at least one contact pair and **e** a histogram of the number of unique contact pairs with all other schools in the network including all school years (i.e. that shown in panel **a**). for all schools (grey) dots, secondary schools (mean age ≥11 years, red circles), and primary schools (mean age <11 years, blue, circles). **f** A histogram of the number of schools connected by at least one contact pair and **g** a histogram of the number of unique contact pairs with all other schools in the network including all school years (grey), the network including only secondary school years (blue) and the network including only primary school years (red).

We constructed a set of seven networks of schools using individual-level de-identified data of pupils attending state-funded schools in England. Links between schools were defined by the number of unique contact opportunities (pupil to pupil) formed through shared households. First, we constructed a network with schools fully open (all pupils attending school) and included 21,583 schools, attended by 4.6 million primary school children and 3.4 million secondary school children in attendance, living at 4.9 million unique addresses (Fig. 1).

**Fig. 2:**
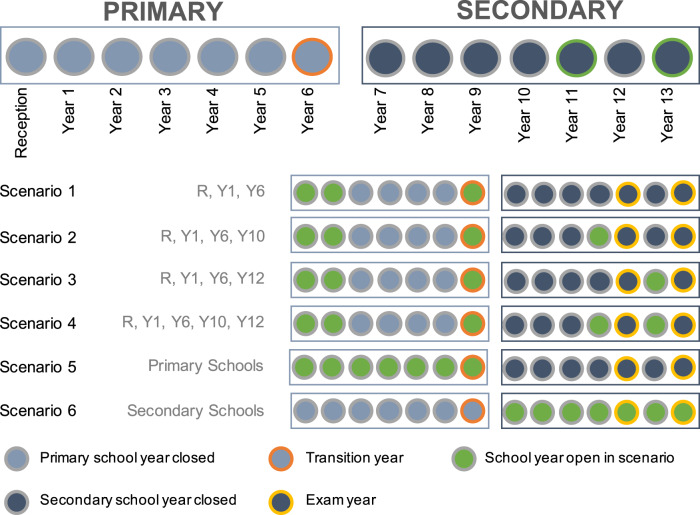
Breakdown of school years in England and reopening scenarios evaluated. Circles represent school years and each row shows a different reopening scenario. Circles are coloured green to indicate inclusion in each scenario. Circles outlined in orange represent a transition year, circles outlined in yellow represent an exam year.

The remaining six networks each represented a reopening scenario relevant to policy in England, illustrated in Fig. 2. In each scenario different combinations of year-groups return to school: early-years education (Reception and Year 1, i.e. 4–6-year-olds) and time-sensitive groups in transition, e.g. through exam certifications or transitional years (Year 6, i.e. 10–11-year-olds, Year 10, i.e. 14–15-year-olds and Year 12, i.e. 16–17-year-olds)^19^. These contained between 21 and 100% of all schools and between 35 and 66% of all households (Table 1).

**Table 1:** The number of schools open and households with children attending school in each school reopening scenario.

Scenario	Number of schools	% of all schools	Number of households	% of all households
1| R, Y1, Y6	17,953	83	1,728,173	37
2| R, Y1, Y6, Y10	21,438	99	2,211,384	45
3| R, Y1, Y6, Y12	19,982	93	1,926,090	39
4| R, Y1, Y6, Y10, Y12	21,480	>99	2,381,729	48
5| Primary schools (R–Y6)	17,984	83	3,267,414	66
6| Secondary schools (Y7–Y13)	4555	21	2,627,640	53
All schools	21,583	100	4,927,163	100

With schools fully open the mean unweighted degree of the schools in the network (average number of schools each school is connected to) was 25 with a maximum of 400. The mean number of contact pairs to all other schools was 184 with a maximum of 2045 (Fig. 1). Secondary schools were more connected to the network with higher mean degree, 65 schools, and weighted degree, 480 contact pairs. Primary schools were less connected with mean degree and weighted degrees of 16 schools and 113 contact pairs, respectively and a maximum degree of 127 schools and weighted degree of 806 contact pairs.

With only primary school years open (i.e. secondary years did not attend school) the mean degree reduced to 6 schools and mean weighted degree to 22 contact pairs. When only secondary school years were open, the mean degree and weighted degree reduced to 22 schools and 103 contact pairs respectively.

### Degree distributions of the transmission probability network

From the contact networks, we estimated the probability of transmission between each pair of schools to assign as edge weights in a transmission probability network for each reopening scenario. With all schools fully open, the mean weighted degree of the transmission probability network (i.e., the mean expected number of schools infected by any individual school) varied between 0.42 for *R* of 1.1, to 3.6 for an *R* of 1.5. The school with the highest weighted degree varied between 4.7 to 35.5 for *R* of 1.1 and 1.5, respectively.

**Fig. 3:**
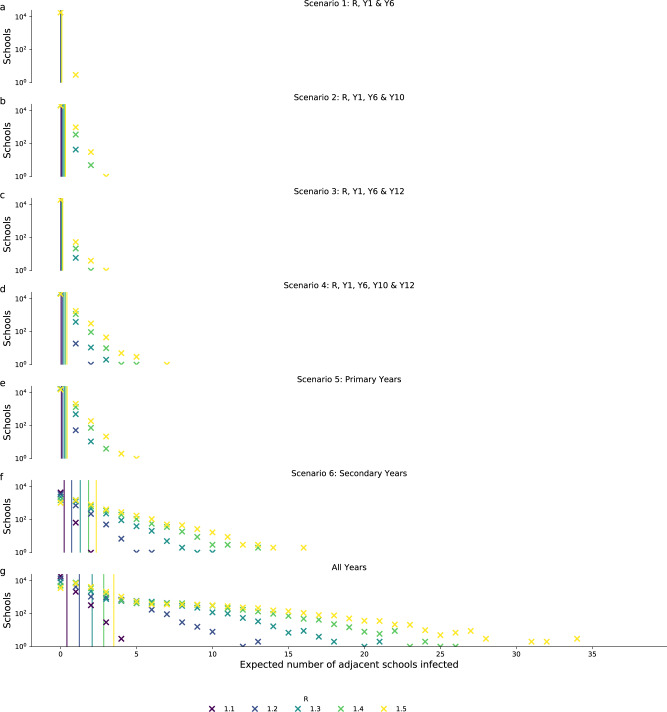
The expected number of schools infected by each school. Weighted degree distribution of the transmission probability network for each of the reopening scenarios considered for *R* values of 1.1–1.5. Panels **a**–**f** show reopening scenarios 1–6, respectively, and panel **g** shows the network with all school years in attendance. Vertical lines show the mean degree for each value of *R*.

When the network was modified to only include pupils from certain years the mean degrees decreased (Fig. 3). Scenario 1 (Reception and Years 1 and 6) had the lowest mean weighted degree (0.01–0.09) for all values of *R*, suggesting that on average each school had ~1–10% chance of infecting one other school. The maximum weighted degree ranged between 0.13 and 1.2, i.e. if an outbreak occurred in the most connected school, it would be expected to infect 1.2 other schools with *R* of 1.5. Scenario 6 (opening secondary schools only) had the highest mean weighted degree, 0.26–2.6 across values of *R* 1.1 to 1.5 suggesting that even at low *R* (1.1) there would be approximately a 25% chance, on average, of infecting a second school and at high *R* (1.5) each school would on average infect 2 or 3 schools during an outbreak. After scenario 6, scenario 5 (primary schools only) had the highest mean degree, between 0.05 and 0.45. Scenarios 2–4, which all combined some partial opening of primary and secondary schools, had relatively similar degree distributions to that of fully opening only, primary schools (Table 2). Of these, scenario 3 (Reception and Years 1, 6 and 12) had the lowest mean degree for each value of *R*, between 0.01 and 0.15.

**Table 2:** Weighted degree of the transmission network and largest components of the binary outbreak networks.

Scenario	*R*	Weighted degree of transmission probability network			Households in largest component of binary outbreak networks			Schools in largest component binary outbreak networks		
		Median	Mean	Max	Median	Low	High	Median	Low	High
1: R, Y1, Y6	1.1	0.00	0.01	0.13	630	596	889	3	2	4
	1.2	0.02	0.02	0.39	843	672	1154	4	3	5
	1.3	0.03	0.05	0.67	1023	800	1350	5	4	7
	1.4	0.04	0.07	0.95	1313	1000	1893	7	5	9
	1.5	0.06	0.09	1.20	1631	1214	2390	9	7	12
2: R, Y1, Y6, Y10	1.1	0.02	0.03	0.27	1009	828	1389	5	4	6
	1.2	0.06	0.10	0.87	2154	1569	3778	10	8	17
	1.3	0.12	0.18	1.60	4790	3262	8693	23	17	40
	1.4	0.17	0.26	2.37	12,529	7940	21,818	66	41	114
	1.5	0.23	0.34	3.11	29,517	21,151	52,983	171	112	272
3: R, Y1, Y6, Y12	1.1	0.01	0.01	0.30	1166	1166	1408	3	3	5
	1.2	0.02	0.04	0.98	1491	1166	2078	6	5	9
	1.3	0.04	0.08	1.82	2173	1651	3685	10	8	16
	1.4	0.07	0.11	2.71	4047	2686	6355	19	13	32
	1.5	0.09	0.15	3.58	7245	4402	13,766	36	22	71
4: R, Y1, Y6, Y10, Y12	1.1	0.02	0.04	0.66	1732	1366	2396	6	5	8
	1.2	0.08	0.12	2.13	4853	3301	7513	18	14	32
	1.3	0.14	0.22	3.91	25,024	14,540	42,580	114	70	186
	1.4	0.21	0.33	5.74	174,846	87,381	226,272	852	427	1139
	1.5	0.27	0.44	7.50	327,433	291,536	403,243	1760	1544	2228
5: Primary years	1.1	0.02	0.05	0.60	1956	1540	2853	6	4	8
	1.2	0.08	0.15	1.46	5366	3947	9253	16	12	26
	1.3	0.14	0.26	2.61	19,639	13,524	32,166	59	38	90
	1.4	0.22	0.36	3.87	55,770	36,732	106,602	167	105	337
	1.5	0.29	0.45	5.10	126,561	76,626	229,320	418	257	768
6: Secondary years	1.1	0.19	0.26	2.21	44,644	26,654	96,454	50	30	106
	1.2	0.60	0.81	6.46	718,224	639,961	887,222	960	842	1212
	1.3	1.08	1.43	10.60	1,588,903	1,299,424	1,859,976	2341	1916	2758
	1.4	1.57	2.04	13.98	2,221,866	2,098,546	2,345,972	3449	3225	3635
	1.5	2.02	2.60	16.91	2,450,215	2,314,264	2,502,364	3904	3658	3998

Median, mean and maximum weighted degree on the transmission probability network (expected number of schools infected by each school) and median range (low and high) of the 90% Credible Interval over 1000 realisations of the binary outbreak network.

### Connected components of binary outbreak networks

Using the transmission probability networks, we generated 1000 realisations of binary outbreak networks for each scenario, where the edges between schools were weighted either 1, with probability equal to the transmission network, or 0. If schools were linked by an edge of weight 1, transmission occurred between the schools in that realisation, edges of weight 0 indicated no transmission between the schools they linked. Connected components on these networks formed groups of schools that would be infected in an outbreak initiated in the same group, for that realisation.

**Fig. 4:**
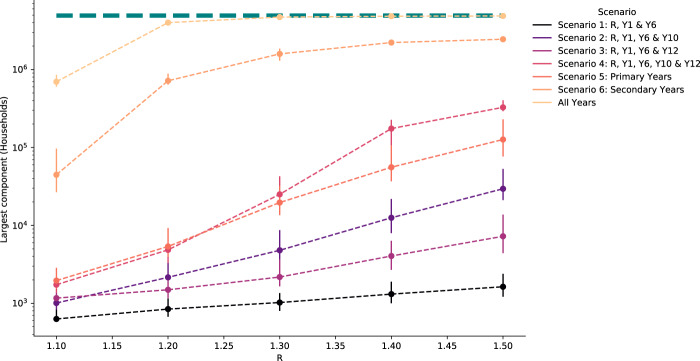
Largest components of the binary outbreak networks. The number of households with children attending a school in each largest connected component of the binary transmission networks (estimated potential outbreak cluster size) generated from transmission probability networks for school reopening scenarios. The points show the median and error bars show the 90% credible intervals for 1000 realisations of binary outbreak networks. The green dashed line shows the total number of households in the school system (4,927,163 households).

**Fig. 5:**
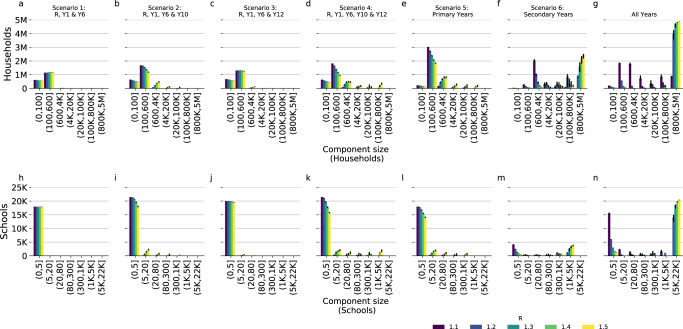
Connected component distributions. The distribution of component sizes of the binary outbreak networks generated for school reopening scenarios and *R* values of 1.1–1.5 (indicated by colour). By households, i.e. the number of households in a component size in each bin, panels **a**–**f** show reopening scenarios 1–6, respectively, and panel **g** shows the network with all school years in attendance. By school, i.e. the number of schools in a component size in each bin, panels **h**–**m** show reopening scenarios 1–6, respectively, and panel n shows the network with all school years in attendance. The bars show the median and error bars show 90% credible intervals for 1000 realisations of binary outbreak networks.

The number of schools in the largest connected component, that is, the number of schools in the largest connected part of the network, increased with *R* for each scenario, increasing the number of households at risk (Fig. 4, Supplementary Fig. 1) For scenario 1 (Reception, Year 1, Year 6), the median largest components simulated ranged between 3 and 9 schools or 630 and 16,031 households across *R* values considered, and there were very few exceeding 10 schools in each realisation (Fig. 5), these connected components typically represented fewer than 1000 households in total.

Adding either of secondary school years 10 or 12 to the network (scenarios 2 and 3) increased the largest connected component size considerably. The size of the largest component was comparable to scenario 1 at a low *R* of 1.1, with a median largest component size of <6 schools for all 5 scenarios. However, the largest connected components for realisations at *R* of 1.5 reached many tens of schools for scenarios 2 and 3 (171 and 36, respectively, compared to 9 for scenario 1) and thousands more households (29,517 and 7245, respectively, compared to 1631 for scenario 1). Adding both Years 10 and 12 had similar largest component size to scenarios 1–3 at 1.1 (6 schools and 1732 households), however, the largest component at 1.5 was much larger than the other scenarios affecting 1760 schools and 327,433 households. Opening only primary school years (scenario 5) resulted in comparable largest component sizes to scenario 4 at lower values of *R* but at *R* of 1.5 resulted in a median largest component of less than a third of schools (median of 418) and less than half as many households (median of 126,561). Largest components were consistently larger when only secondary schools were included in the network, with a median of 50 schools and 44,644 households with an *R* of 1.1 increasing to 3904 schools and 2,450,215 households at an *R* of 1.5 which accounts for 85% of schools and 93% of households.

Despite the increase in largest component size at higher values of *R*, for scenarios 1–5, the substantial majority of schools remained in small components of <5 schools, even with *R* at 1.5: 17,909 (>99% of schools in the network), 18,024 (84%), 19,442 (97%), 15,716 (73%), 14,130 (79%) for scenarios 1–5, respectively. Whereas for scenario 6, where all secondary school years return, only 538 (12% of schools in the network) schools formed components of <5 schools.

## Discussion

Our results suggest that allowing schools to open with a small selection of school years may only present a small risk of transmission between schools and, consequently, the households of school children. The analysis also highlights the difference in risk posed by secondary schools relative to primary schools, where reopening even a small subset of secondary school years (Years 10 and 12) increases the connectivity between schools considerably, whereas opening all primary schools resulted in lower connectivity in the network. Furthermore, opening secondary schools alone resulted in the highest connectivity of all the partial reopening scenarios evaluated.

Recent studies showed that outbreaks in primary schools were smaller than in secondary schools in the same area^20^ and that older children might pose a greater risk of onwards transmission in households than younger ones^21^. In combination, these studies suggest that primary schools contribute less to community infections than secondary schools and support the prioritisation of allowing primary schools to open^17^, although if children in secondary schools were better able to practice physical distancing than primary schools, this could act to counterbalance the additional risk. Under the assumption that primary school children transmit the virus less efficiently than older school children^15^, the difference between the scenarios of reopening either primary or secondary schools would be expected to be greater than what we found. In the extreme case where primary school children were not able to transmit the virus at all, the scenario of reopening all years would be the same as reopening only secondary schools. Our assumption is that transmission between school-aged children is sufficient to sustain an outbreak within a school, i.e. *R* > 1. Although there is some evidence of transmission within schools^20,22^ and that closing schools reduced the growth rate of the epidemic^23^, other studies have shown that transmission in schools did not contribute greatly to the overall epidemic prior to closure^24,25^. Since reopening in September there has been mixed evidence of transmission of SARS-CoV-2 in schools^26,27^. However, because evidence of school outbreaks is largely based on passive case detection, the true risk of school transmission may be substantially under-reported as children have a lower risk of developing symptoms after infection. Moreover, UK prevalence surveys show 11–18-year-olds routinely have the second-highest prevalence after 18–29-year-olds. Further, school children are estimated to be several times more likely to introduce infection into the household than adults—a rate which has increased since schools reopened in September^16^, suggesting that transmission in schools may have been an important factor in driving the outbreak since school reopening. Consensus on this matter remains elusive^28^, and our results should therefore be considered in light of the most recent available evidence to the reader.

Although we found that varying the reproduction number within the schools, *R*, had a substantial impact on the number of households in the largest potential outbreak cluster (indicated by the largest component), there was little impact on the results for the vast majority of schools’ component sizes, suggesting that particular parts of the network were more closely connected than the rest of the network. This could translate to particular geographical areas being disproportionately affected following the reopening of schools. Increasing *R* also had some impact on the weighted degree distribution of the transmission probability network, suggesting that in that case the virus may spread more effectively across connected components even if the eventual outbreak cluster size remained similar. This may impact the effectiveness of targeted interventions, as identifying a school outbreak before an outbreak in an adjacent school has been seeded may become more challenging. This is analogous to challenges in contact tracing due to pre-symptomatic infection^29,30^.

Our network focusses on transmission in schools and households between school-aged children and aims to provide insight into the capacity for transmission within schools and households to develop into large outbreak clusters involving multiple schools. Further, we cannot account for mixing among children from different schools or households occurring outside of school contexts^12^. The data from which the network was constructed, included only state-funded schools in England with children coded as school years Reception to Year 13 in official data. The addition of independent schools would increase the size and possibly the connectivity of the network, however, only 7% of children in England attend an independent school so the impact may be marginal.

Our model presupposes that the expected outbreak risk within the school network is closely related to the risk within the wider community. That is, the risk of an infectious pupil seeding a school outbreak is proportional to the prevalence of infection in the community. Therefore, the transmission risks associated with opening schools would be expected to increase as prevalence in the surrounding community increases.

The way we quantified the probability of transmission between schools assumed that each school outbreak reached its theoretical unmitigated final size, this may not occur if interventions, such as targeted school or class closure are introduced. For example, closure of schools when a small number of cases are reported could be an effective means to curb transmission^31,32^ early on, however, to the knowledge of the authors, the effectiveness of such reactive closures is yet to be quantified in the context of SARS-COV-2. This framework also implies a well-mixed contact network within each school, final sizes are likely to be smaller due to preferential mixing within school years, classes and by gender^33–35^. In addition, if schools implement social bubbles to introduce community structure in the contact network and therefore reduce the probability of a school-wide outbreak^36^. This is partly reflected in the low values of *R* that have been chosen relative to those estimated early in the outbreak of 2.0–3.1)^37^ but our estimates of the number of households impacted may still be an overestimate compared to any real situation which would include mitigation measures (e.g., improved hand hygiene and use of face masks) and reactive interventions in response to cases detected in schools.

Our framework assumes no presence of immunity, however, there is evidence of immunity to SARS-COV-2 in children^16^. The true immunity in schools is likely to vary both by region and between schools, however, the resolution of data on immunity in England is poor and certainly cannot be resolved at a school level. Similarly, the reproduction number was assumed to be invariant between schools, this approach was chosen to maintain the parsimony of the approach, as modelling internal transmission dynamics of individual schools would considerably increase the complexity. In light of these simplifications, our results should be interpreted as the maximal risk posed by transmission within and between schools.

We assumed child-to-child transmission within households occurs with probability *q* = 0.15, which is consistent with estimates of the household secondary attack rate^38,39^. To assess the robustness of the results to this assumption, we re-ran the analysis with *q* = 0.3 and *q* = 0.08 (Supplementary Figs. 2–5), and although the sizes of the connected components changed, the relative impact of scenarios remained comparable to the main analysis. In the absence of more robust evidence, however, we cannot rule out that transmission between children might be different from general transmission patterns to a degree that would fundamentally affect our results.

Our analysis provides insight into the potential for school-based and household-based contacts between children to combine to create long chains of transmission which could result in infections within many thousands of households. We highlight that the potential contribution of schools to transmission varies substantially between the tested scenarios. Reintroducing primary school years had much lower risk of transmission between schools than secondary school years. We also highlight that maintaining restrictions on contact between children within schools to ensure a low within-school reproduction number may be highly influential, as the rate of transmission between schools increases rapidly with *R* on some parts of the network. Furthermore, such restrictions may be essential for suppressing transmission. While our results should not be considered as realistic epidemiological projections, our simulations provide an indication of the relative impact of each scenario, using highly resolved schools data. Further analysis using this network may provide more precise guidance, particularly on reactive school closure strategies in the event of detecting a school outbreak, where the network itself may serve as a tool to aid targeted interventions. If detailed projections were desired, the framework could be extended to include within-school contact structure, however, this would greatly increase the network size and therefore computational effort required. The principles highlighted in our analyses are not constrained to SARS-CoV-2 and may be considered when evaluating interventions for any epidemic in which children are known to transmit infection.

Our results are directly applicable to the school system in England. Although the network properties of school systems around the world may vary, we anticipate these results would be qualitatively similar in other settings with broadly comparable education frameworks.

## Methods

### Data

Individual-level de-identified data of pupils attending state-funded schools in England was provided by the UK Department for Education (DfE) under a formal data-sharing agreement. The use of this data was also reviewed and approved (Ref: 22476) by the London School of Hygiene & Tropical Medicine Research Ethics Committee. The data includes an entry for each pupil for each institution they attend, Unique Reference Number (URN) for the school, school postcode, pupil’s postcode and pupil’s address, collected between September and December 2019. We combined the student’s postcode and address to assign a household code for each group of pupils that were found to live at the same address, where we assume each individual address operates as a single household for social distancing purposes. We tested this method by comparing the assigned codes to official unique address codes provided in the data for 53% of the pupils. We found that of these, 99.8% of households with more than one pupil were correctly identified as a single household and 0.2% of households were mistakenly merged with another household (Supplementary Table 1). Using our assigned household code, we were able to estimate the number of unique contacts between each pair of schools. For each pupil, we have included only institutions coded as the pupil’s current main school and have excluded pupils listed as boarders (those who are resident at their school during term time). More details of data cleaning are included in Supplementary Note 1.

### Reopening scenarios

Typically, there are 14 school years in the English school system (Fig. 2), which each run from September to September. Children enter Reception aged 4 and complete 7 years of primary school leaving Year 6 aged 11. They transition to secondary school into Year 7 where all pupils are expected to complete 5 years of secondary education (until the age of 16). At this point, children are able to leave school or progress to further education (FE), which may be in the same institution as other secondary school years or a separate institution offering only FE courses.

There are exceptions to this two/three institution framework, where some schools offer a different subset of school years (for example the first 3 years of primary education). For this analysis all reopening scenarios are assumed to operate on a school year basis, hence assuming that all children from the appropriate years return regardless of the nature of their institution.

We considered six reopening scenarios relevant to policy in England, illustrated in Fig. 2. In each scenario different combinations of year-groups return to school: early-years education (Reception and Year 1, i.e. 4–6-year-olds) and time-sensitive groups in transition, e.g. through exam certifications or transitional years (Year 6, i.e. 10–11-year-olds, Year 10, i.e. 14–15-year-olds and Year 12, i.e. 16–17-year-olds)^19^.

### A network of transmission between schools

**Fig. 6:**
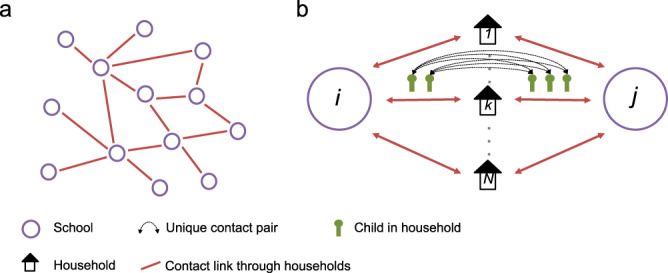
Schematic to demonstrate the principle of a network of schools linked by households. **a** A network of schools constructed such that schools are connected when contact is made between pupils of different schools within a household. **b** The strength of contact between schools is quantified by calculating the number of unique contact pairs (one child in each school). The number of pairs per household is the product of the number of children who attend school *i* and the number of children who attend school *j*. The total number of unique pairs is the sum of unique pairs over all, *N*, households, *k*, with children attending both school *i* and *j*.

We used the data to construct a network of schools linked through households. Each edge on the network of schools is weighted by the number of unique contacts between schools that occur through shared households. For example, if in a given household, 2 children attend school *i* and 2 children attend school *j*, this corresponds to 4 unique contacts between school *i* and school *j*. The total number of unique contacts between schools *i* and *j*, denoted by *C*_*ij*_, is the sum of unique contacts over all households (Fig. 6). Concretely,

1 $$\begin{array}{*{20}{c}} {C_{ij} = \mathop {\sum }\limits_k n_{k,i}n_{k,j}} \end{array}$$

Where *n*_*k,i*_ is the number of children in household *k* who attend school *i*.

**Fig. 7:**
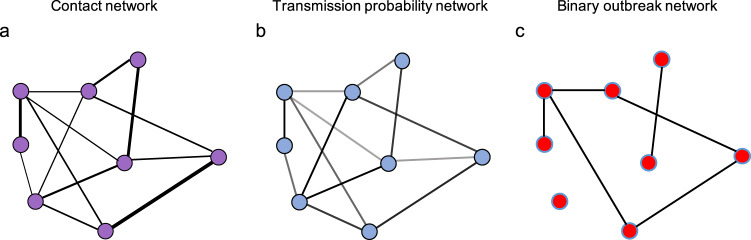
How contact, transmission and binary outbreak networks relate to each other. **a** A schematic of a contact network, the width of the edges shows the number of unique contact pairs between schools. **b** A schematic of a transmission probability network calculated from the contact network; the shading of the edges shows the relative probability of transmission between schools. **c** A schematic of a realisation of a binary outbreak network, where edges are weighted 1 with probability given by the equivalent edge in the transmission network—indicating transmission between schools, or 0 otherwise. Blue highlighted nodes show those in the largest connected component.

From this network, we created a transmission probability network (Fig. 7) where we estimated the probability of transmission between schools *i* and *j* (*P*_trans*,ij*_).

We defined transmission between schools as an outbreak in one school leading to an outbreak in an adjacent school on the network. We simplify within-household transmission such that only direct transmission between contact pairs occurs (neglecting the potential for transmission through other members of a household) and hence approximate the transmission probability between schools through a single contact pair as.

2 $$\begin{array}{*{20}{c}} {P_{{\mathrm{ob}}}P_{{\mathrm{inf}},j}q} \end{array}$$

where *P*_ob_ is the probability of an outbreak in school *i* given one infection, *P*_inf*,j*_ is the probability of a child in school *j* being infected and *q* is the probability of transmission between children in the same household.

The probability of transmission between schools *j* and *i* through all contact pairs can be approximated as

3 $$\begin{array}{*{20}{c}} {P_{{\mathrm{trans}},ij} = 1 - \left( {1 - P_{{\mathrm{ob}}}P_{{\mathrm{inf}},j}q} \right)^{C_{ij}}} \end{array}$$

We estimated the probability of an outbreak *P*_ob_ to be:

4 $$\begin{array}{*{20}{c}} {P_{{\mathrm{ob}}} = 1 - 1/R} \end{array}$$

which reflects a geometric distributed contact rate within the school^40^. Here, *R* is the within-school reproduction number, the average number of secondary infections in a single school from an index case where all others are susceptible. This statistic differs from the reproduction number in the general population as it only includes secondary cases infected within school, which are the result of transmission from only school-aged children.

We assumed homogeneous mixing within the school population. We then approximated the probability of a student in school *j* being infected $P_j^I$ based on the expected final size^40^ of an outbreak with within-school reproduction number *R*,

5 $$\begin{array}{*{20}{c}} {P_{{\mathrm{inf}},j} = Z_\infty = 1 - e^{ - RZ_\infty }} \end{array}$$

where *Z*_*∞*_ is the final outbreak size as a proportion of the school population.

We set *q*, the per-contact probability of transmission between children in the same household, to 0.15 (consistent with estimates of household secondary attack rate of SARS-CoV-2^38^).

We repeated the analysis for a range of within-school*R* values between 1.1 and 1.5, leading to outbreak size between 18 and 58% of school children, broadly spanning the range of reported outbreak sizes of COVID-19 in schools^20,22^.

For each scenario, we assumed all pupils within the years specified attended school and contributed to transmission. We assumed that pupils outside of the specified years did not attend school and therefore did not contribute to transmission. To simulate this condition, we constructed a network using only data of pupils in the specified years.

### Evaluating the network

To summarise how the potential of transmitting to adjacent schools in the network varies with *R* (within school) and the reopening scenario we calculated the distribution of the weighted degree *D* of the transmission network (the distribution of the expected number of schools infected through households by each school) for each scenario, where the weighted degree of school *i*, *D*_*i*_ was defined as:

6 $$\begin{array}{*{20}{c}} {D_i = \mathop {\sum }\limits_j C_{ij}} \end{array}$$

To summarise the potential spread of the virus across the network of schools, we sampled instances of binary outbreak networks, where transmission between each pair of schools either occurs (edge weight of 1) or does not occur (edge weight 0) (Fig. 7).

Since transmission probabilities are reciprocal, the eventual number of schools in any outbreak cluster can be defined as a connected component of the outbreak network (i.e. all schools are connected by edges equal to 1). For a particular school *i*, the schools in the same connected component are those that would be infected in an outbreak seeded at that school (*i*). The same schools are those in which a seeded outbreak would eventually infect this school (*i*). Hence the distribution of the connected components gives an indication of expected outbreak size and therefore risk posed to and by individual schools in the network.

Schools vary in size considerably, with large differences between secondary and primary schools. To reflect the size of outbreaks in terms of the number of households at risk, we calculated the number of households with children attending schools within each connected component in the network. Specifically, we calculated the number of unique households with children attending the schools in each component (in the appropriate years for each scenario). To summarise the risk of larger outbreak clusters, we present the distribution of the number of households associated with each connected component.

All analysis in this study was performed using python 3.7.3^41^. Network analysis was performed using NetworkX 2.4^42^.

### Reporting summary

Further information on experimental design is available in the Nature Research Reporting Summary linked to this paper.

## Supplementary information

Supplementary Information

Peer Review File

Reporting Summary

## Data Availability

The data that support the findings of this study were made available from UK Department for Education (DfE) but restrictions apply to the availability of these data, which were used under license for the current study, and so are not publicly available. Due to the sensitive nature of the data, they can only be made available by DfE through a data-sharing agreement directly with the user.

## Code availability

The code used for this analysis is available from https://github.com/jdmunday/SchoolHouseholdNetworksCOVID and archived in a permanent respository^43^.
