# Supplementary material for: Implications of the school-household network structure on SARS-CoV-2 transmission under school reopening strategies in England
Source: Nat Commun. 2021 Mar 29;12:1942. doi: 10.1038/s41467-021-22213-0 (PMC8007691; doi:10.1038/s41467-021-22213-0)
Supplement: Supplementary file 1 — Supplementary Information [file 41467_2021_22213_MOESM1_ESM.docx]

Supplementary Information for *Implications of the school-household network structure on SARS-CoV-2 transmission under different school reopening strategies in England*:

***Supplementary Figures***


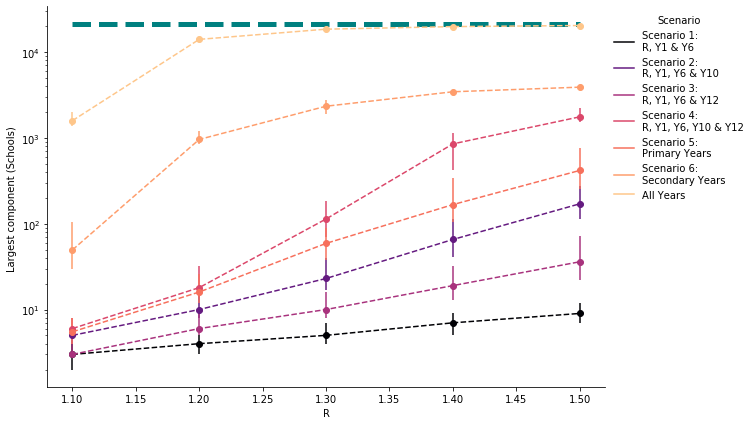


**Supplementary Figure 1. Largest components of the binary outbreak networks (schools).** The number of schools in each largest connected component of the binary transmission networks generated from transmission probability networks for school reopening scenarios. The points show the median and error bars show the 90% credible intervals for 1000 realisations of binary outbreak networks. The green dashed line shows the total number of schools in the school system.


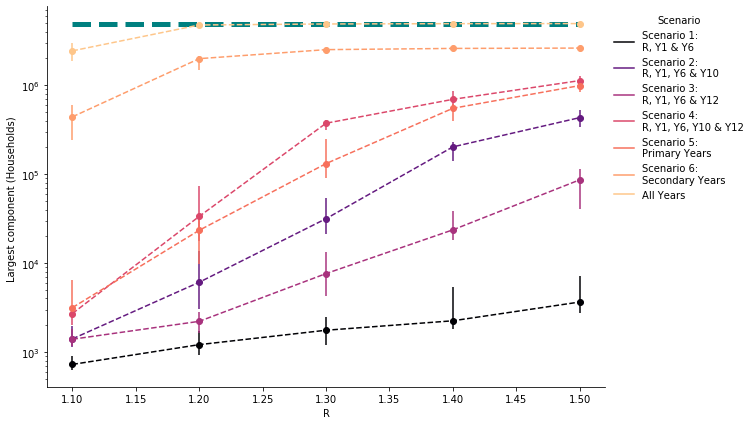


**Supplementary Figure 2. Largest components of the binary outbreak networks (Sensitivity analysis with probability of within-household transmission, q = 0.3).** The number of households with children attending a school in each largest connected component of the binary transmission networks (estimated potential outbreak cluster size) generated from transmission probability networks for school reopening scenarios. The points show the median and error bars show the 90% credible intervals for 1000 realisations of binary outbreak networks. The green dashed line shows the total number of households in the school system.


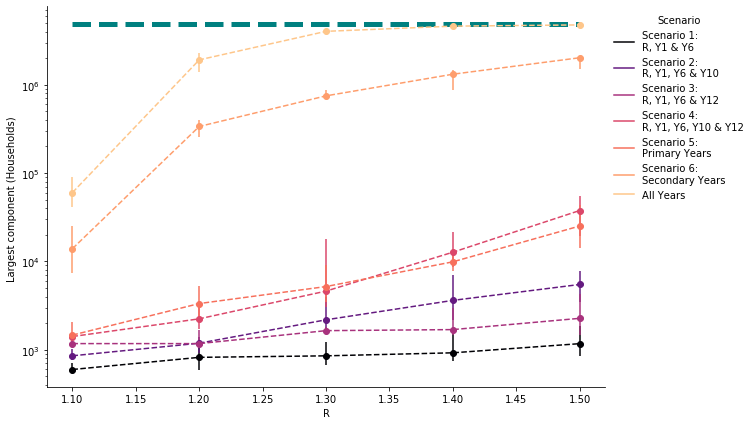


**Supplementary Figure 3. Largest components of the binary outbreak networks (Sensitivity analysis with probability of within-household transmission, q = 0.08).** The number of households with children attending a school in each largest connected component of the binary transmission networks (estimated potential outbreak cluster size) generated from transmission probability networks for school reopening scenarios. The points show the median and error bars show the 90% credible intervals for 1000 realisations of binary outbreak networks. The green dashed line shows the total number of households in the school system.


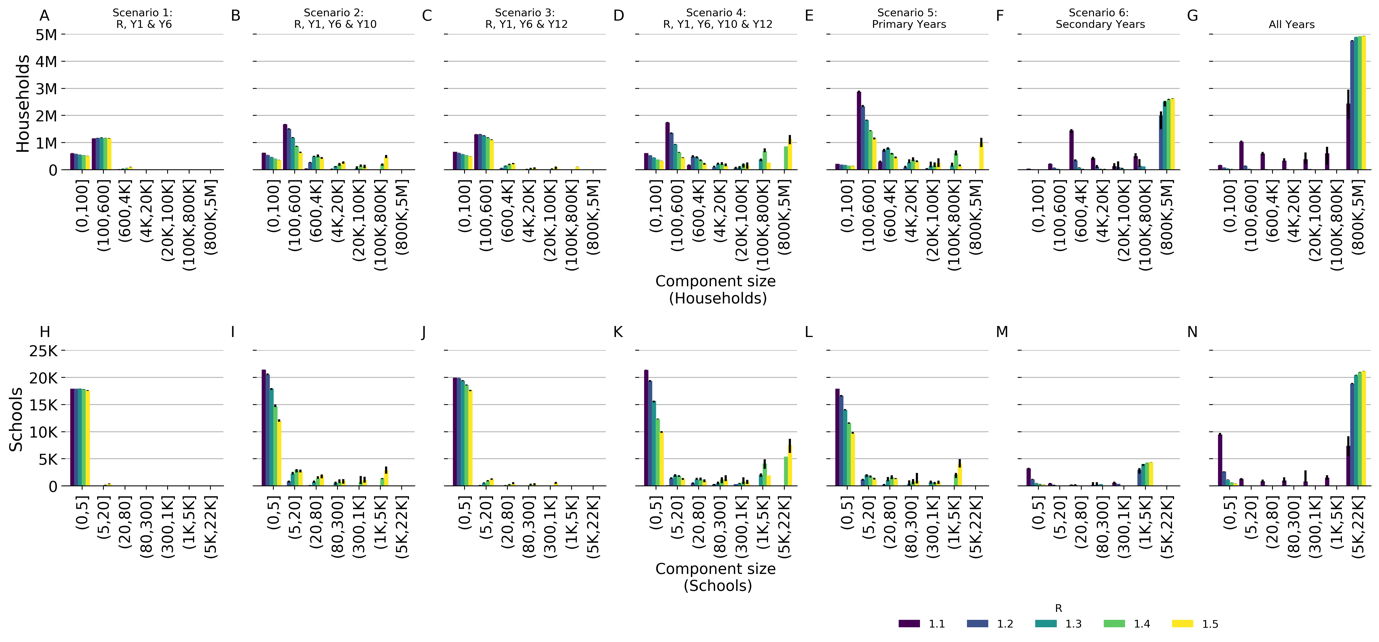


**Supplementary Figure 4. Connected component distributions. (Sensitivity analysis with probability of within-household transmission, q = 0.3).** The distribution of component sizes of the binary outbreak networks generated for school reopening scenarios and *R* values of 1.1 to 1.5 (indicated by colour). By households (A-G), i.e. the number of households in a component size in each bin and by school (H-N). The bars show the median and error bars show 90% credible intervals for 1000 realisations of binary outbreak networks.


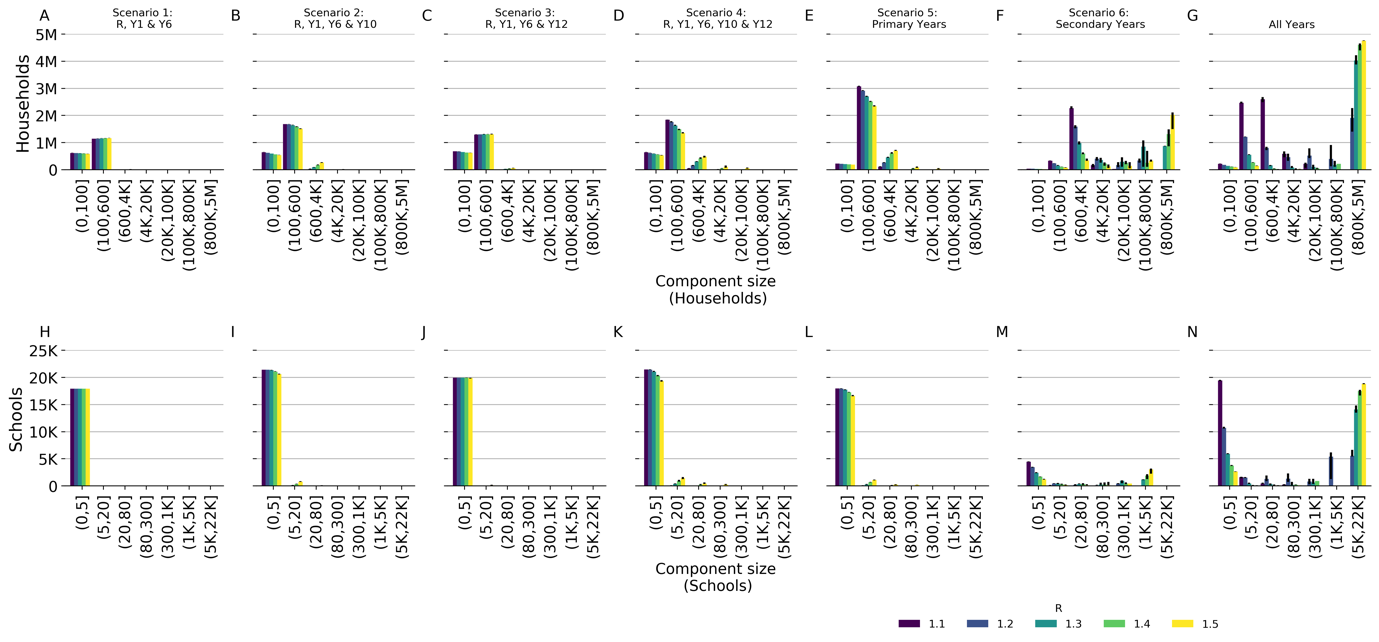


**Supplementary Figure 4. Connected component distributions. (Sensitivity analysis with probability of within-household transmission, q = 0.08).** The distribution of component sizes of the binary outbreak networks generated for school reopening scenarios and *R* values of 1.1 to 1.5 (indicated by colour). By households (A-G), i.e. the number of households in a component size in each bin and by school (H-N). The bars show the median and error bars show 90% credible intervals for 1000 realisations of binary outbreak networks.

***Supplementary Tables***

**Supplementary Table 1.** **Address matching validation.** Household addresses with a UK standard unique property reference number (UPRN), against households identified by postcode and address matching. 53% of households (2.6m of 4.9m) had a UPRN.

|  | Number of true unique households by UPRN (N=2627601) |
| --- | --- |
| 1 household correctly identified | 2621685 (99.78%) |
| 2 households incorrectly merged | 5902 (0.22%) |
| 3 households incorrectly merged | 14 (<0.001%) |

***Supplementary Notes***

**Supplementary Note 1.**

Approximately half of students had a unique postal address code for their residence. We evaluated our address matching against this code for the students that had one. Table S1 shows the proportion of households that were correctly identified as a single household (99.78%) and the proportion of households that were mistakenly merged with either 1 or 2 others 0.22% and 0.001% respectively).
