# Supplementary material for: Implications of the school-household network structure on SARS-CoV-2 transmission under school reopening strategies in England
Source: Nat Commun. 2021 Mar 29;12:1942. doi: 10.1038/s41467-021-22213-0 (PMC8007691; doi:10.1038/s41467-021-22213-0)
Supplement: Supplementary file 3 — Reporting Summary [file 41467_2021_22213_MOESM3_ESM.pdf]

nature research

Corresponding author(s): James Munday

Last updated by author(s): Feb 23, 2021

Reporting Summary

Nature Research wishes to improve the reproducibility of the work that we publish. This form provides structure for consistency and transparency in reporting. For further information on Nature Research policies, see our Editorial Policies and the Editorial Policy Checklist.

Statistics

For all statistical analyses, confirm that the following items are present in the figure legend, table legend, main text, or Methods section.

n/a	Confirmed	
☐	☒	The exact sample size (*n*) for each experimental group/condition, given as a discrete number and unit of measurement
☐	☒	A statement on whether measurements were taken from distinct samples or whether the same sample was measured repeatedly
☒	☐	The statistical test(s) used AND whether they are one- or two-sided<br>*Only common tests should be described solely by name; describe more complex techniques in the Methods section.*
☒	☐	A description of all covariates tested
☐	☒	A description of any assumptions or corrections, such as tests of normality and adjustment for multiple comparisons
☐	☒	A full description of the statistical parameters including central tendency (e.g. means) or other basic estimates (e.g. regression coefficient) AND variation (e.g. standard deviation) or associated estimates of uncertainty (e.g. confidence intervals)
☒	☐	For null hypothesis testing, the test statistic (e.g. *F*, *t*, *r*) with confidence intervals, effect sizes, degrees of freedom and *P* value noted<br>*Give P values as exact values whenever suitable.*
☒	☐	For Bayesian analysis, information on the choice of priors and Markov chain Monte Carlo settings
☒	☐	For hierarchical and complex designs, identification of the appropriate level for tests and full reporting of outcomes
☒	☐	Estimates of effect sizes (e.g. Cohen's *d*, Pearson's *r*), indicating how they were calculated

Our web collection on statistics for biologists contains articles on many of the points above.

Software and code

Policy information about availability of computer code

Data collection	No data collection was performed as part of this study
Data analysis	We used python 3.7.3 for all analysis, network analysis was performed using networkx 2.4, the code can be found at https://github.com/jdmunday/SchoolHouseholdNetworksCOVID

For manuscripts utilizing custom algorithms or software that are central to the research but not yet described in published literature, software must be made available to editors and reviewers. We strongly encourage code deposition in a community repository (e.g. GitHub). See the Nature Research guidelines for submitting code & software for further information.

Data

Policy information about availability of data

All manuscripts must include a data availability statement. This statement should provide the following information, where applicable:

- Accession codes, unique identifiers, or web links for publicly available datasets
- A list of figures that have associated raw data
- A description of any restrictions on data availability

The data that support the findings of this study were made available from UK Department for Education (DfE) but restrictions apply to the availability of these data, which were used under license for the current study, and so are not publicly available. Due to the sensitive nature of the data, they can only be made available by DfE through a data sharing agreement directly with the user.

Field-specific reporting

Please select the one below that is the best fit for your research. If you are not sure, read the appropriate sections before making your selection.

- [x] Life sciences
- [ ] Behavioural & social sciences
- [ ] Ecological, evolutionary & environmental sciences

For a reference copy of the document with all sections, see nature.com/documents/nr-reporting-summary-flat.pdf

Life sciences study design

All studies must disclose on these points even when the disclosure is negative.

Sample size	7,905,826 (This is a near complete sample of school children attending state schools in England)
Data exclusions	Before commencing analysis we excluded childen under the following conditions: We excluded children coded as bording at school as they are not expected to contribute to transmission. Children in households with > 20 children as this was deemed as improbable and likely to be incorrectly coded (<1% of pupils).
Replication	This is a mathematical modeling study. Replication is not applicable, all variability in the simulations are captured in the monte carlo anlysis and presented in the plots and tables.
Randomization	The data used in this analysis constitutes a near complete sample of school children in the UK, randomization is therefore not applicable.
Blinding	This is a mathematical modeling study, no experiments or trials were carried out therefore blinding is not applicable.

Reporting for specific materials, systems and methods

We require information from authors about some types of materials, experimental systems and methods used in many studies. Here, indicate whether each material, system or method listed is relevant to your study. If you are not sure if a list item applies to your research, read the appropriate section before selecting a response.

Materials & experimental systems

n/a	Involved in the study	
x		Antibodies
x		Eukaryotic cell lines
x		Palaeontology and archaeology
x		Animals and other organisms
	x	Human research participants
x		Clinical data
x		Dual use research of concern

Methods

n/a	Involved in the study	
x		ChIP-seq
x		Flow cytometry
x		MRI-based neuroimaging

Human research participants

Policy information about studies involving human research participants

Population characteristics	School aged children attending state funded schools in England: Home address, age, gender, school attended, school address, school year.
Recruitment	The data was extracted from the Department for Education pupil records as entered by schools in Autumn 2019. This is a near complete sample of children in state funded schools in England no significant biases are expected due to extremely high coverage of the underlying population.
Ethics oversight	The use of this data was reviewed and approved by London School of Hygiene and tropical medicine ethics committee ref: 22476

Note that full information on the approval of the study protocol must also be provided in the manuscript.
