# Supplementary material for: Implications of the school-household network structure on SARS-CoV-2 transmission under school reopening strategies in England
Source: Nat Commun. 2021 Mar 29;12:1942. doi: 10.1038/s41467-021-22213-0 (PMC8007691; doi:10.1038/s41467-021-22213-0)
Supplement: Supplementary file 2 — Peer Review File [file 41467_2021_22213_MOESM2_ESM.pdf]

Editorial note: This manuscript has been previously reviewed at another journal that is not operating a transparent peer review scheme. This document only contains reviewer comments and rebuttal letters for versions considered at *Nature Communications*.

REVIEWERS' COMMENTS

Reviewer #1 (Remarks to the Author):

Thank you for responding to my comments. I am happy with the revised version.

Reviewer #2 (Remarks to the Author):

Authors have sufficiently addressed my earlier comments. I am happy to recommend publication.
